# Prospective gating control for highly efficient cardio-respiratory synchronised short and constant TR MRI in the mouse

**DOI:** 10.1016/j.mri.2018.06.017

**Published:** 2018-11

**Authors:** Paul Kinchesh, Stuart Gilchrist, John S. Beech, Ana L. Gomes, Veerle Kersemans, Robert G. Newman, Borivoj Vojnovic, Philip D. Allen, Michael Brady, Ruth J. Muschel, Sean C. Smart

**Affiliations:** Cancer Research UK and Medical Research Council Oxford Institute for Radiation Oncology, Department of Oncology, University of Oxford, United Kingdom

**Keywords:** Rapid, Fast, Prospective, Gating, Reacquisition, Mouse

## Abstract

**Purpose:**

Cardiac and respiratory motion derived image artefacts are reduced when data are acquired with cardiac and respiratory synchronisation. Where steady state imaging techniques are required in small animals, synchronisation is most commonly performed using retrospective gating techniques but these invoke an inherent time penalty. This paper reports the development of prospective gating techniques for cardiac and respiratory motion desensitised MRI with significantly reduced minimum scan time compared to retrospective gating.

**Methods:**

Prospective gating incorporating the automatic reacquisition of data corrupted by motion at the entry to each breath was implemented in short TR 3D spoiled gradient echo imaging. Motion sensitivity was examined over the whole mouse body for scans performed without gating, with respiratory gating, and with cardio-respiratory gating. The gating methods were performed with and without automatic reacquisition of motion corrupted data immediately after completion of the same breath. Prospective cardio-respiratory gating, with acquisition of 64 k-space lines per cardiac R-wave, was used to enable whole body DCE-MRI in the mouse.

**Results:**

Prospective cardio-respiratory gating enabled high fidelity steady state imaging of physiologically mobile organs such as the heart and lung. The automatic reacquisition of data corrupted by motion at the entry to each breath minimised respiratory motion artefact and enabled a highly efficient data capture that was adaptive to changes in the inter-breath interval. Prospective cardio-respiratory gating control enabled DCE-MRI to be performed over the whole mouse body with the acquisition of successive image volumes every 12–15 s at 422 μm isotropic resolution.

**Conclusions:**

Highly efficient cardio-respiratory motion desensitised steady state MRI can be performed in small animals with prospective synchronisation, centre-out phase-encode ordering, and the automatic reacquisition of data corrupted by motion at the entry to each breath. The method presented is robust against spontaneous changes in the breathing rate. Steady state imaging with prospective cardio-respiratory gating is much more efficient than with retrospective gating, and enables the examination of rapidly changing systems such as those found when using DCE-MRI.

## 1 Introduction

In small animal MRI, retrospective gating has become the standard means of performing steady state maintained scanning in conjunction with cardio-respiratory motion desensitisation [[1], [2], [3]]. Each k-space line is acquired repeatedly and the temporal position of each acquisition within the physiological cycles is recorded using, for example, electrode derived electrocardiogram (ECG) measurements [1], or by the use of the MRI signals themselves [2, 3]. The acquired data are subsequently reordered for alignment with the physiological cycles prior to image reconstruction. The advantage is that user set-up can be relatively straightforward since it is not necessary to detect the R-wave from ECG signals in the presence of imaging gradients, which is considered to be problematic [[4], [5], [6], [7], [8], [9]]. The method is, however, relatively inefficient since each k-space line has to be acquired repeatedly over the physiological cycles. Retrospective cardio-respiratory gating (CR-gating) requires each k-space line to be repeated for a time period that is at least the maximum expected duration of the cardiac cycle, and this provides a fundamental limit to imaging speed in all instances where the acquisition of an additional CINE dimension is not a specific requirement.

Prospective gating is defined, in this report, according to the ‘spin-conditioned’ mode of Ehman et al. [10] where the scanner runs continuously at a predefined constant repetition time (TR) and performs different actions according to the instantaneous level of a binary control signal that may be high or low. In this case the scanner either performs a data acquisition element, or a dummy element during which data are not acquired, in response to the state of the control signal. RF and gradient pulsing are performed according to the defined TR throughout the scan in order to maintain the forced equilibrium steady state magnetisation amplitudes and, where applicable, phases.

Prospective cardiac synchronisation in small animal MRI has traditionally been performed with R-wave triggered acquisition combined with respiratory blanking such that only those R-waves that occur during the relatively motion free inter-breath period are used for imaging. In its simplest implementation one k-space line is acquired at each heartbeat during the inter-breath period, and TR is dictated by the instantaneous R-R interval and by the delay that occurs for each breath during which RF and gradient pulsing is suspended. Severe signal amplitude modulation occurs due to the large variation in TR which results in image ghosting. This problem has been largely alleviated by dummy scanning whilst awaiting the next inter-breath R-wave that is deemed useful for imaging [11]. This removes the TR dependence on the breath duration, and a greatly improved level of signal stabilisation is achieved.

Variants that enable a shorter TR for CINE or segmented acquisitions require the number of repetitions to be performed within each R-R interval to be specified in advance, and an acquisition block is triggered by the detection of each R-wave [12]. These scan modes still feature mixed duration TR periods with fixed TR during the data acquisition block and a variable TR that is introduced whilst waiting for the next R-wave trigger. The signal modulations that persist can present as image ghosting and/or intensity modulations which make the approach unsuitable in instances that require the steady state magnetization to be held within specific and stringent limits during a fixed TR throughout the entire scan, such as variable flip angle *T*_1_ mapping and balanced SSFP.

In this approach, the number of inter-breath R-waves that are used for data acquisition is also specified in advance. Respiration artefacts have been minimised by setting the imaging period to be shorter than the shortest observed respiratory period [11]. This compromises scan efficiency and the scan is not rendered entirely immune from data corruption due to an earlier than anticipated breath.

The short TR prospective gating method reported here enables the spin system magnetisation to be maintained in the steady state indefinitely until the control signal for data acquisition that is derived from a physiological event (such as an R-wave) is registered by the scanner. In this implementation, a block of N k-space lines is acquired whenever the control signal for data acquisition is registered. These data are typically acquired in a centre-out phase encode order when cardiac synchronisation is performed so as to ensure the intensity information is most closely aligned with the R-wave. As such, the imaging time for prospective CR-gating is reduced when compared to retrospective gating methods by a factor N, the number of k-space lines that are acquired in each CR-gated data block. Data acquired at the onset of each breath are identified as being potentially corrupted by the breath, and are automatically reacquired after the end of the same breath. This allows an adaptive control of the respiratory gating with data acquisition throughout the inter-breath period regardless of its duration and provides a method that is robust against spontaneous changes in the breathing rate. The rapid imaging described enables the examination of changes that occur in DCE-MRI whilst maintaining good image fidelity and high throughput operation.

## 2 Methods

### 2.1 In vivo preparation and gating control

**Fig. 1:**
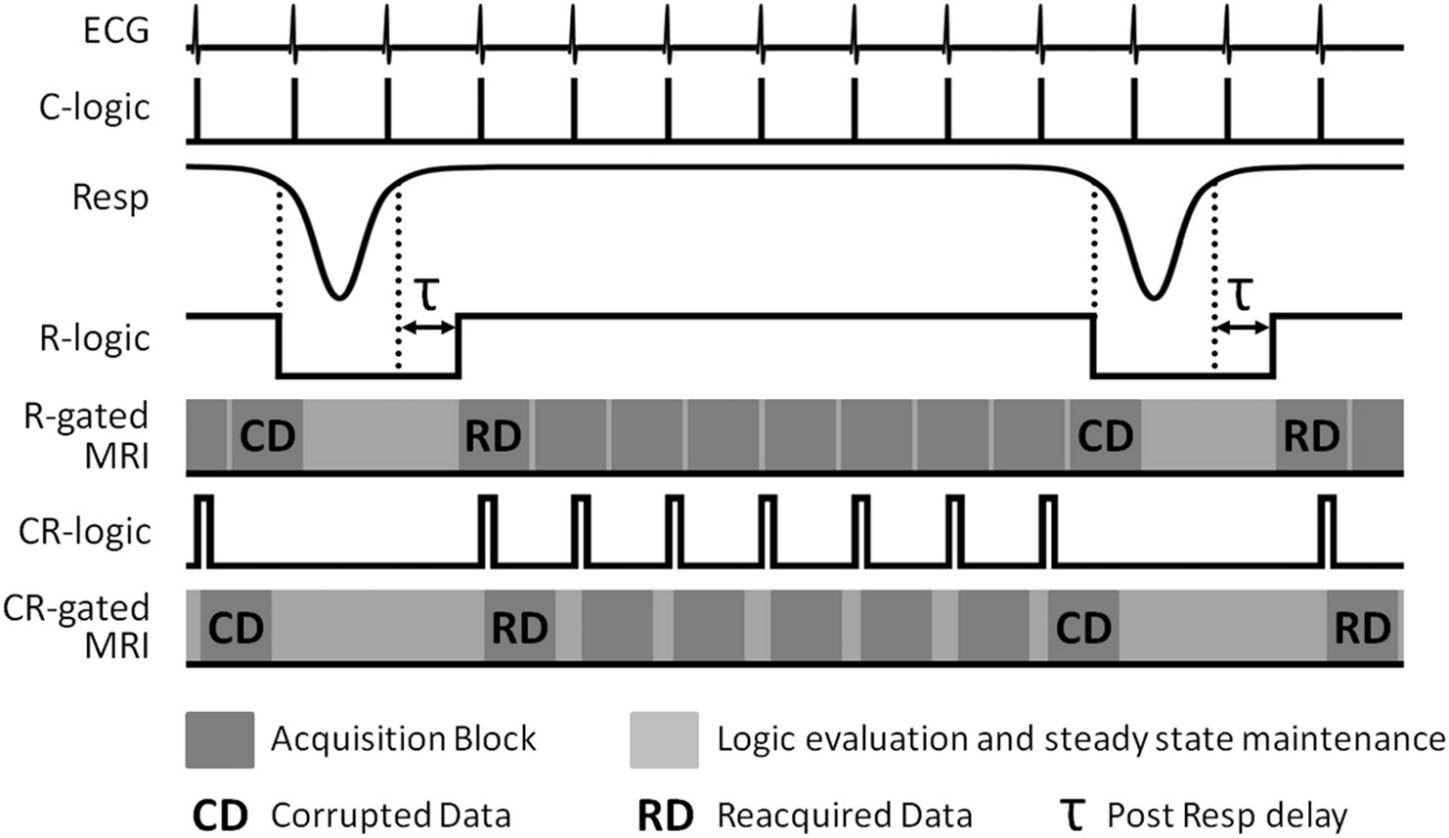
Diagrammatic representation of respiration gated (R-gated) and cardio-respiratory gated (CR-gated) MRI schemes. Threshold levels are set on the amplified and filtered ECG and respiration (Resp) analogue voltages to generate the C-logic and R-logic control signals respectively. The R-logic control signal is evaluated for R-gated scanning. A user-variable post breath delay (τ) is used to ensure that motion artefact is minimised from the trailing portion of the breath. Only the C-logic signals that occur during the R-logic high level gate are selected to generate the CR-logic control signal which is evaluated for CR-gated scanning. The duration of each CR-logic high signal is extended to last longer than one TR to ensure detection when the CR-logic state is polled once every TR, but must not exceed the duration of the acquisition block otherwise it will cause the next block to be acquired prematurely. The use of threshold level respiration detection decrees that each breath starts before it is detected. In the diagram a single respiration corrupted data acquisition block (marked CD) is automatically reacquired as soon as each breath completes (marked RD) to reduce artefact from motion during the onset of each breath.

All animal studies were performed in accordance with the UK Animals (Scientific Procedures) Act of 1986 under licences approved by the UK Home Office and with the approval of the University of Oxford ethical review committee. CBA mice (Charles River) were anaesthetised with 3% isoflurane in air and placed supine on a cradle for MRI where anaesthesia was maintained with 1–2% isoflurane in a 1:5 O_2_:air mixture. Rectal temperature was monitored with an optical system (ACS-P4-N-62SC and OTP-M, Opsens Inc., Quebec, Canada) that provided feedback to a twisted pair resistive heating system developed for MR compatible homeothermic maintenance [13]. Respiration was monitored using a pneumatic balloon (VX010, Viomedex Ltd., UK) positioned against the animal's chest and coupled to a pressure transducer. The problems associated with detection of the R-wave from ECG signals in the presence of imaging gradients were overcome by using platinum subdermal needle electrodes F-E2-12 (Grass Technologies, Natus Neurology, RI) placed subcutaneously in the chest to form only a small induction loop that is connected directly to shielded cabling. The raw respiration and ECG signals were interfaced to a physiological recording system (MP150, Biopac Systems Inc., CA using DA100C and ECG100C-MRI amplifiers respectively) and the amplified and filtered signals were processed with the custom-built hardware detailed in the Appendix to generate logic control signals for the prospective respiration gating (R-gating) and cardio-respiratory gating (CR-gating) strategies of Fig. 1.

The level of motion corruption in images acquired using prospective gating was evaluated for different gating schemes in four mice, whilst whole body DCE-MRI with cardio-respiratory synchronisation was demonstrated in one mouse although further data are available [14].

### 2.2 MRI

MRI was performed on a 7.0 T 210 mm horizontal bore VNMRS preclinical imaging system equipped with 120 mm bore gradient insert (Varian Inc., CA). RF transmission and reception was performed with a 100 mm long 25 mm ID quadrature birdcage coil (Rapid Biomedical GmbH, Germany).

For the examination of image stability as a function of gating control, 3D spoiled gradient echo imaging was performed with TR 2.8 ms, TE 1.0 ms, FOV 108 × 27 × 27 mm^3^, matrix 256 × 64 × 64, gradient spoiling with 128 mT/m for 1.0 ms in all three axes, RF hard pulse duration 16 μs, FA 8° and RF spoiling [15]. Scans were run without gating control, with respiratory gating control and with both cardiac and respiratory gating control. An isoflurane anaesthetized (1–3% in air and/or O_2_) normal healthy mouse takes snatched breaths of about 200 ms duration with a significantly depressed and often variable respiration rate, typically 30–60 breaths per minute depending on the depth and duration of anaesthesia [[2], [3], [4], 11, 16]. The heart rate is moderately depressed and generally lies well within the 400–600 bpm (beats per minute) range, corresponding to an R-R interval of 100–150 ms [[16], [17], [18]]. For respiratory gating control, blocks of 64 k-space lines corresponding to a complete fast phase encode set were acquired in a linear order over 179.2 ms whenever the R-logic control signal state evaluation registered an inter breath period. This was considered appropriate since it enabled acquisition of several blocks within a typical inter breath period. For cardio-respiratory synchronisation, segmented blocks of 32 k-space lines, fitting well within a single R-R interval, were acquired in a centre-out phase encoding order (first segmented block k-lines 0 to 31, second segmented block k-lines -1 to -32), over 89.6 ms whenever the CR-logic control signal state evaluation registered an inter breath R-wave. Dummy scanning was performed for at least 10 s prior to data acquisition to ensure generation of the steady state, and scanning was performed for approximately 8.5 min with 44, 24 and 20 repetitions for the ungated, respiratory gated (R-gated), and cardio-respiratory gated (CR-gated) scans, respectively. The number of repetitions performed for each scan mode was specifically selected to keep the total data acquisition time as uniform as possible since the stability within a fixed period of time is considered to provide the most meaningful comparison between the different gating methods. In order to validate the time series data against a target, a high SNR ‘best representation’ image was formed from the average of the 20 scans acquired with CR-gated control and automatic reacquisition, as this image was expected to show the minimum physiologically derived corruption.

**Fig. 2:**
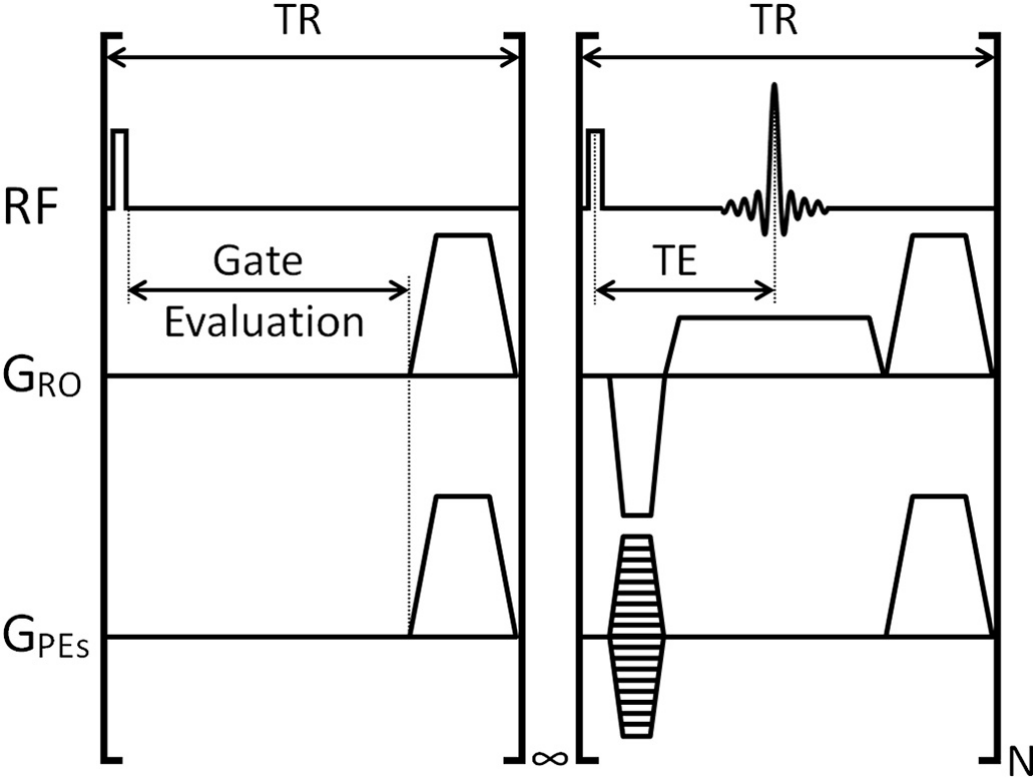
Diagrammatic representation of the scan structure for a prospectively gated constant TR 3D gradient echo scan with RF and gradient spoiling. The gate evaluation loop persists indefinitely until the logic high level of the gating signal is detected whereupon acquisition of a block of N k-lines is invoked.

Prospectively gated scans were structured to accommodate the proprietary gating evaluation dead time as depicted in Fig. 2.

For R-gated and CR-gated control with automatic reacquisition, the two data blocks acquired prior to detection of each breath were reacquired immediately after the same breath. The reacquisition of two data blocks ensures motion corruption upon entry to the breath is minimised because it is entirely possible for the R-logic signal to switch and register a breath immediately after the gate evaluation has informed the scanner to acquire a block of data. Images were reconstructed separately using either the initially acquired data, or the reacquired data, in order to assess the potential of reacquisition. Image stability was examined by visual inspection, intensity measurement for selected ROIs positioned about the body, and by display of the standard deviation of the mean for the time series images that were produced using the different gating control schemes.

For whole body DCE-MRI, 3D spoiled gradient echo imaging was performed with TR 1.5 ms, TE 1.0 ms, FOV 108 × 27 × 27 mm^3^, matrix 256 × 64 × 64, gradient spoiling with 128 mT/m for 1.0 ms in all three axes, RF hard pulse duration 16 μs, FA 5°, and RF spoiling. 64 k-space lines were acquired in a centre-out order (k-lines 0,-1,1,-2,2, …,-31,31,-32) in a time of 96 ms whenever the CR-logic control signal state evaluation registered an inter breath R-wave. 100 repeats of the 3D scan were performed with a blood-pool Gd-contrast agent (Gadospin-P, Viscover, 100 μl) infused via a tail vein cannula over 15 s starting at the beginning of frame 11/100. Data from the two heartbeats acquired prior to detection of each breath were reacquired immediately after the same breath.

## 3 Results and discussion

**Fig. 3:**
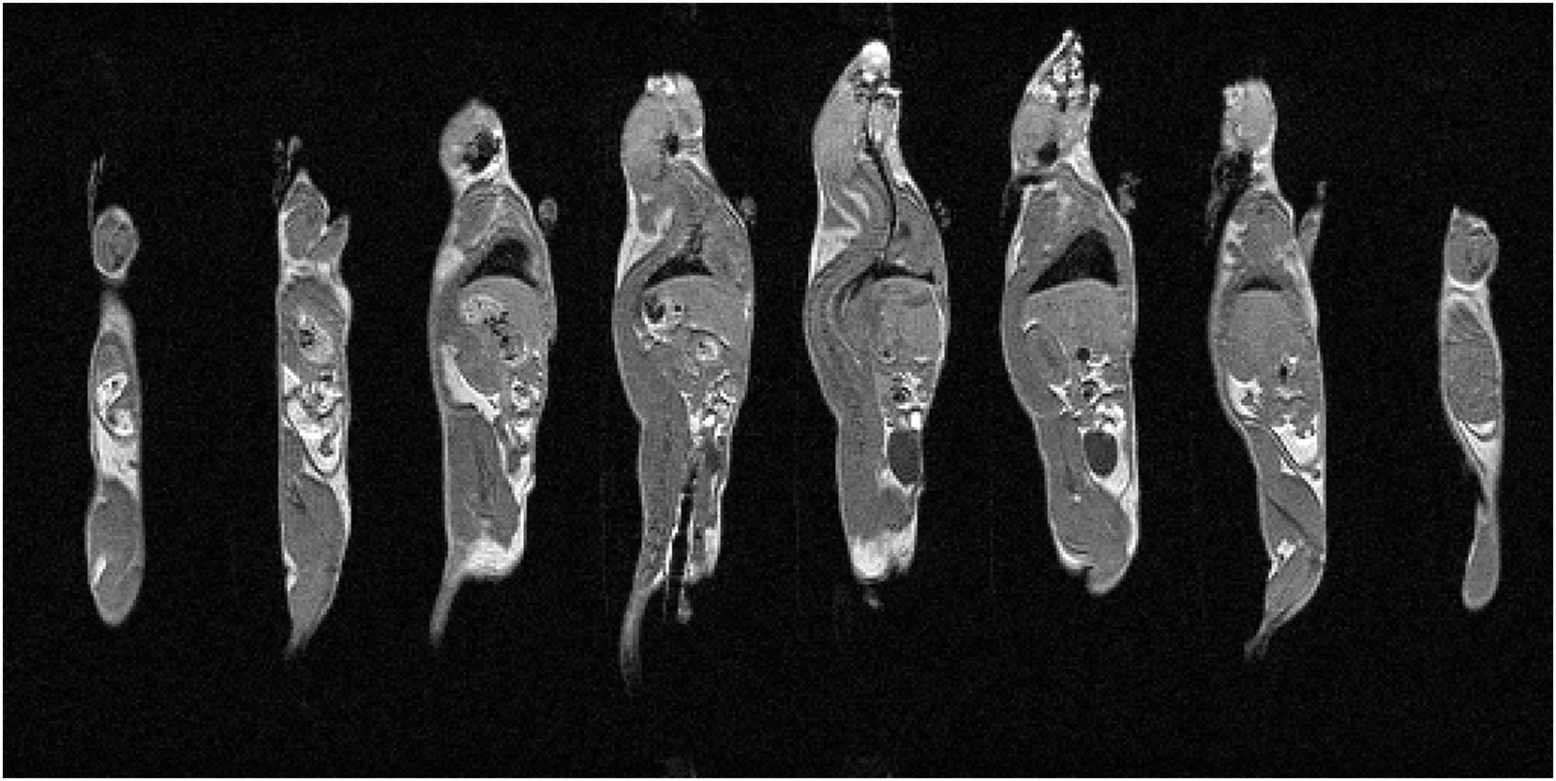
Eight non-contiguous slices taken from a sagittal view through a 3D image volume using prospective CR-gating with automatic reacquisition of respiratory motion corrupted data. The data shown are from mouse 1, repetition 1.

Anatomical *T*_1_-weighted images taken from a 3D image volume acquired using prospective CR-gating with respiratory reacquisition in a scan time of about 25 s are presented in Fig. 3.

Highly detailed anatomical information is seen and inter-organ boundaries such those positioned between liver and lung, liver and kidney, and kidney and muscle are clearly defined. This level of anatomical detail, especially in the abdomen and thorax, was only achievable through the use of cardiac and respiratory synchronisation with the reacquisition of data that were corrupted upon the entry to each breath.

**Fig. 4:**
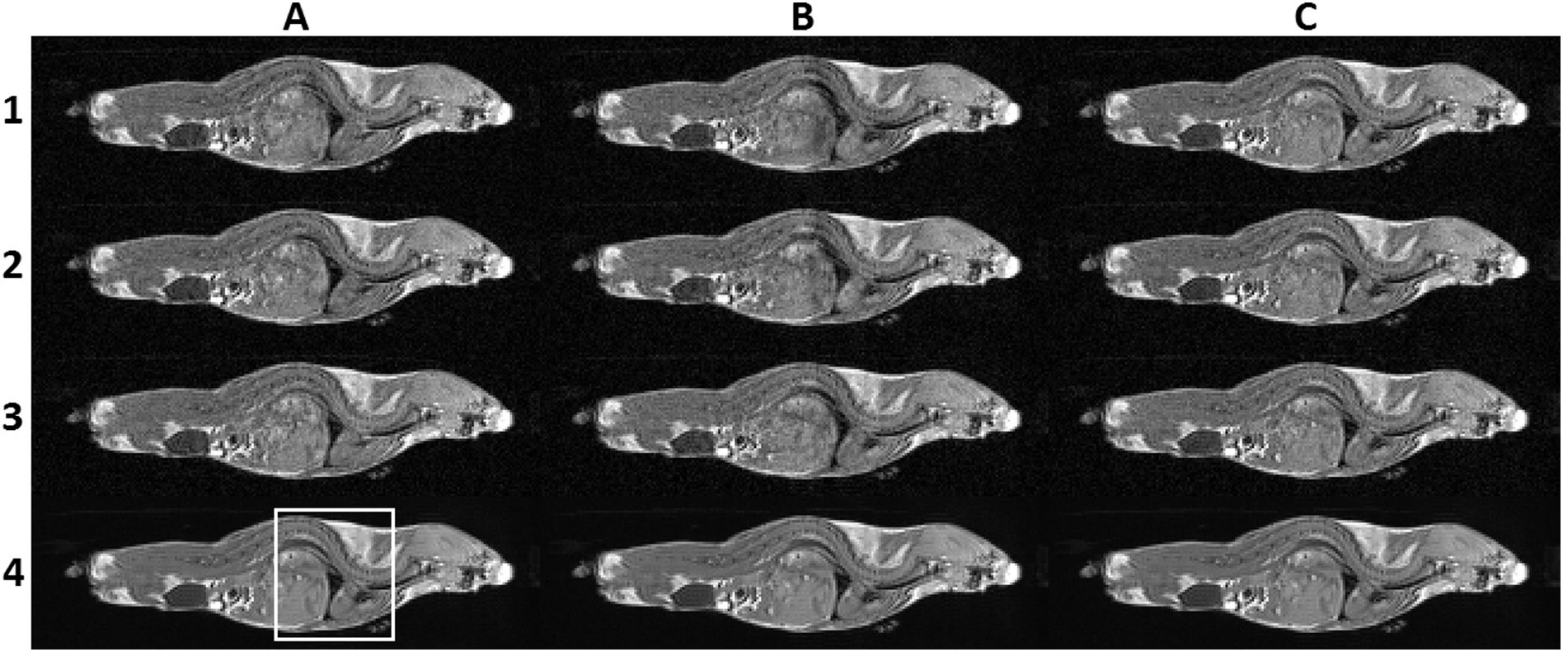
Rows 1–3 show three consecutively acquired images for scans performed with no gating (column A), with CR-gating but no automatic respiratory reacquisition (column B), and for CR-gating with automatic respiratory reacquisition (column C). Row 4 shows the ‘best representation’ image which was produced from the average of the 20 image volumes acquired using cardio-respiratory synchronisation with automatic reacquisition of respiratory motion corrupted data. The boxed region enclosing the liver and heart is expanded for each image in Fig. 5. The data shown is slice 35 from mouse 1 using repetitions 1, 2 and 3 for each of the acquisition schemes which are entirely representative of all the data.

**Fig. 5:**
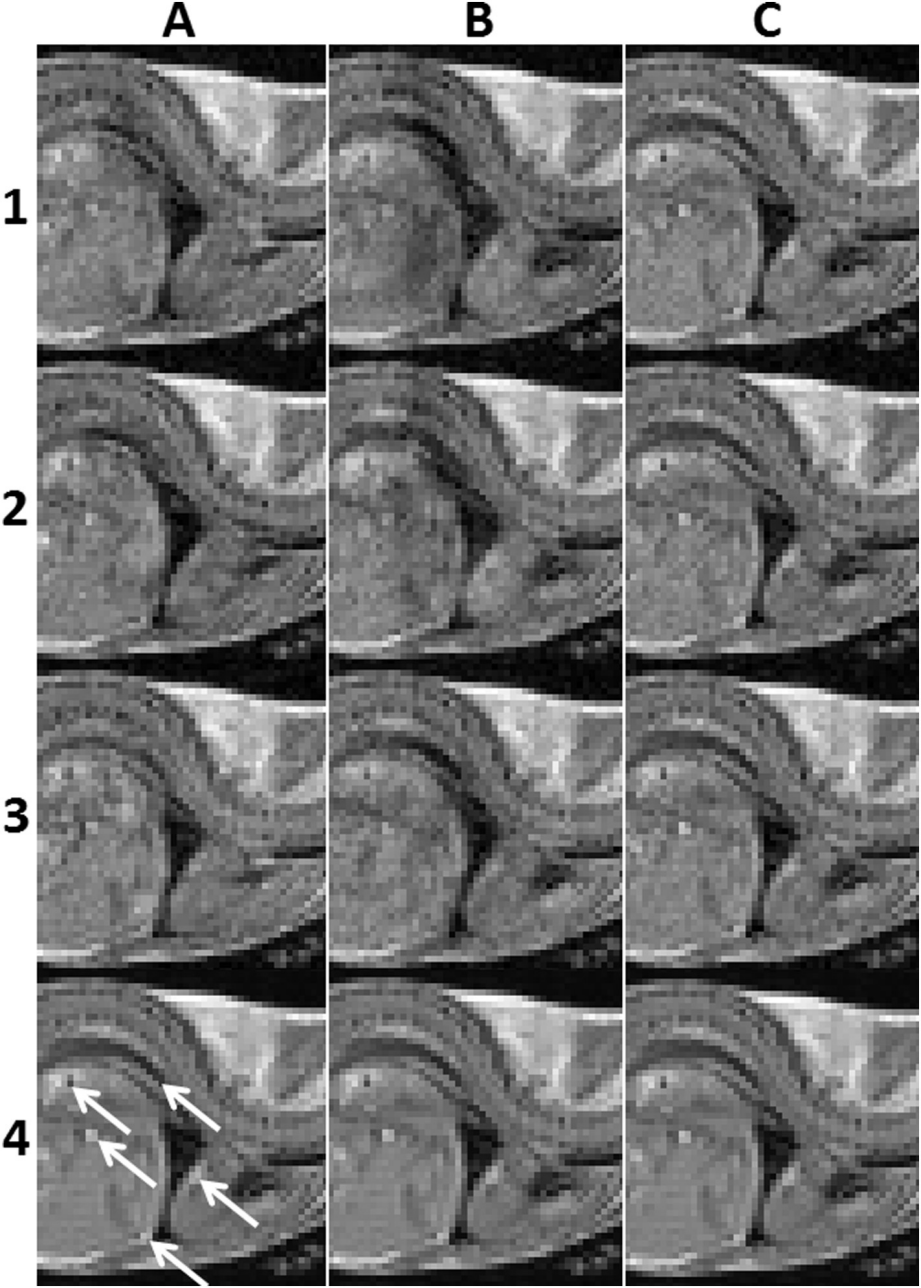
Rows 1–3 show three consecutively acquired images for scans performed with no gating (column A), with CR-gating but no automatic respiratory reacquisition (column B), and for CR-gating with automatic respiratory reacquisition (column C). Row 4 shows the ‘best representation’ image which was produced from the average of the 20 image volumes acquired using cardio-respiratory synchronisation with automatic reacquisition of respiratory motion corrupted data. Arrow tips are used to highlight some example structural features. Column C shows a consistent and faithful detection of the example structural features in rows 1–3 demonstrating improved image stabilisation.

Fig. 4 shows three consecutively acquired frames through the mouse body for images produced without gating, with CR-gating but without respiratory reacquisition, and with CR-gating and respiratory reacquisition. These images are compared with the ‘best representation’ display in the bottom row which was produced from the average of the 20 image volumes acquired using CR-gating with automatic reacquisition of respiratory motion corrupted data. An enlarged view of the boxed region enclosing the liver and heart is expanded for each image in Fig. 5 where the tips of arrow heads are used to highlight several structural features whose sharpness and visibility is compromised without good gating control.

It is readily apparent that severe and temporally unstable image distortions are present in the abdomen and thorax in the absence of gating control (column A, rows 1–3) and in the absence of the automatic reacquisition of data corrupted upon entry to the breath (column B, rows 1–3). With CR-gating control and the automatic reacquisition of the respiratory motion corrupted data the individual image frames (column C, rows 1–3) match the features of the ‘best representation’ image (row 4), as can be seen by the consistent and faithful detection of the example structural features. Improved stability was observed across the entire time course in multiple regions throughout the body with CR-gating and automatic reacquisition.

**Fig. 6:**
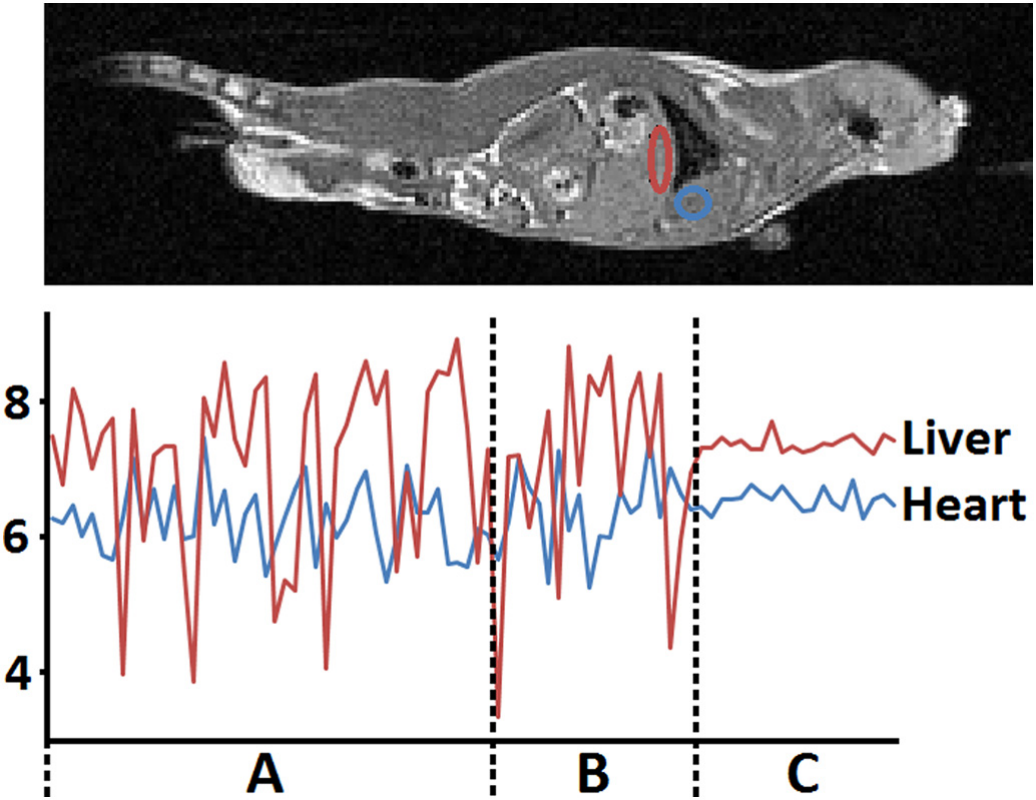
Top. ROI locations in the head-ward liver and heart (mouse 1, slice 28). Bottom. Mean intensity plots (arbitrary units) of the ROIs for the time series images acquired with no gating (44 repetitions, period A), with CR-gating but no automatic reacquisition (20 repetitions, period B), and for CR-gating with automatic reacquisition (20 repetitions, period C).

This stability is further highlighted in the ROI analysis plots for selected organs shown in Fig. 6 which, again shows that a markedly improved stability is given only where CR-gating is used in conjunction with automatic reacquisition.

**Fig. 7:**
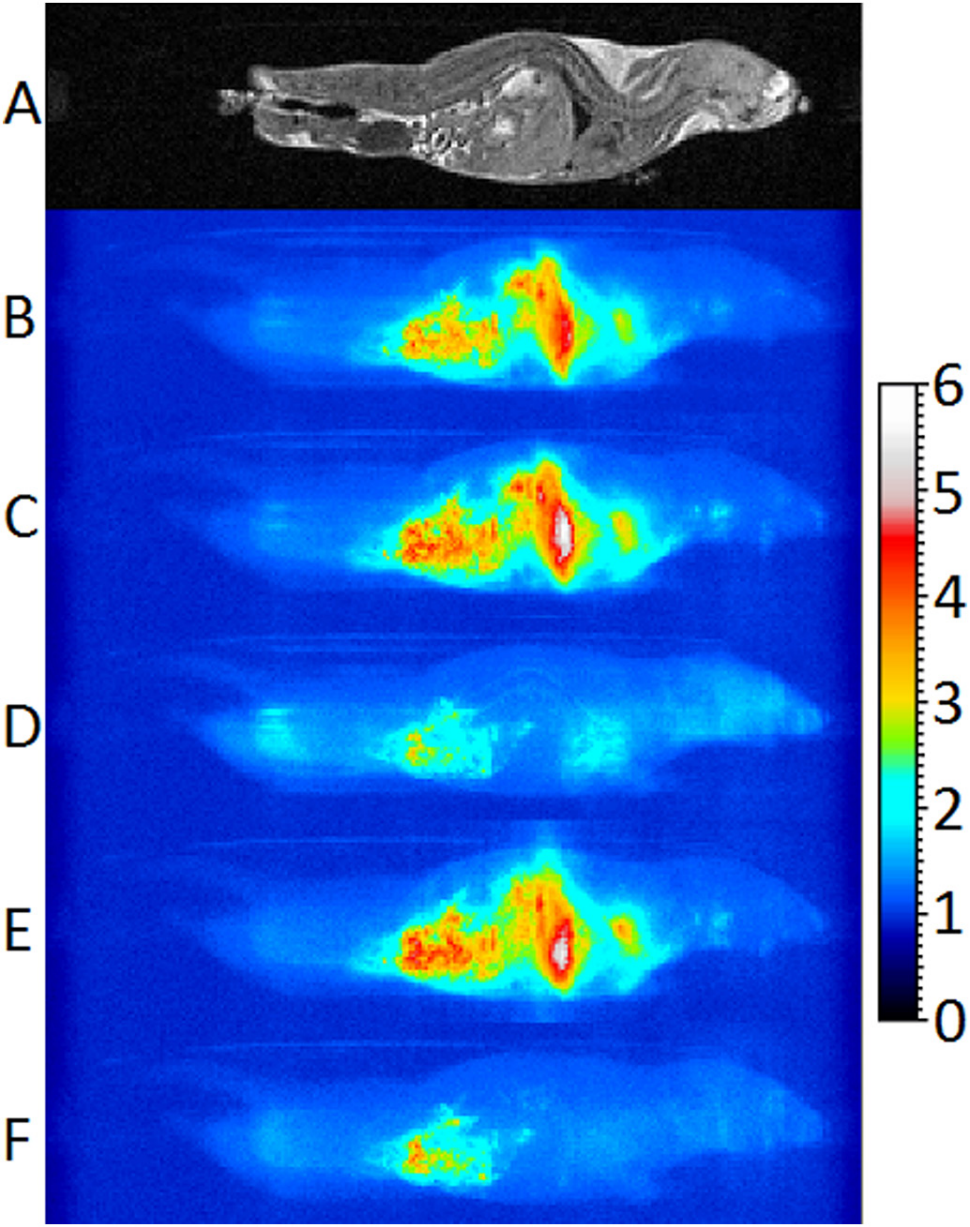
Sagittal image slice and summed sagittal projections of standard deviation maps with uniform scaling in arbitrary units generated from 3D data acquisition repetitions of different scan modes acquired in about 8.5 min of scanning for mouse 1. A: Example central sagittal slice. B-F: Summed sagittal projections of standard deviation maps. B: No gating, 44 reps. C: R-gating without reacquisition, 24 reps. D: R-gating with reacquisition, 24 reps. E: CR-gating without reacquisition, 20 reps. F: CR-gating with reacquisition, 20 reps.

Fig. 7 shows images representing the standard deviation of the mean signal intensity for the time series data acquired over about 8.5 min with: no gating; R-gating but no automatic reacquisition; R-gating with automatic reacquisition; CR-gating but no automatic reacquisition; and CR-gating with automatic reacquisition.

The stability within a fixed period of time is considered to provide the most meaningful comparison between the different gating methods since it is the best way to maintain the number of breaths, heartbeats and amount of gut motion (i.e. the sources of instability) as constant as possible.

In the absence of gating, the instabilities primarily cover the thorax and abdomen (Fig. 7B). Performing R-gating and CR-gating strategies without imposing a shortened inter breath imaging period or implementing the reacquisition scheme essentially show the same level of instabilities in the thorax and abdomen (Fig. 7C and E). Performing R-gating with reacquisition significantly reduces the extent and level of instabilities, restricting them to the gut, where peristalsis derived motions are present, and regions within and surrounding the heart (Fig. 7D). A small instability around the main arteries due to the pulsatile blood flow becomes apparent between the heart and gut. Performing CR-gating with reacquisition removes the instabilities associated with cardiac function (Fig. 7F). A comparison of R-gating with and without reacquisition (Fig. 7C and D) shows a slight loss of stability within the head and hind when image reconstruction uses the reacquired data. We suspect this is because the head and hind are more affected by respiration motion after the snatched breath as the motion propagates through the body. A comparison of CR-gating with and without reacquisition (Fig. 7E and F) shows a much smaller difference. When CR-gating is performed there is frequently an additional delay until the next R-wave which appears to have reduced the effect.

Overall, it is quite clear that respiratory motion presents the largest volume and scale of instability. Respiratory corruption is inevitable if data acquired during the entry to the breath are used in image reconstruction. The lowest standard deviations were measured where cardiac-respiratory synchronisation was used in conjunction with reacquisition of respiratory motion corrupted data.

The use of threshold detection of the breaths requires that any data acquired during the entry to the breath is subject to corruption through motion, and this is shown to be minimised through the use of the reacquisition technique described. Without reacquisition it would be possible to minimise respiration motion artefact by setting a suitable acquisition window that is likely to complete before the next breath. However, this invokes a considerable time penalty if the window is set conservatively and data acquisition during respiration as a result of a shorter than anticipated inter breath interval becomes almost inevitable. Furthermore, the unpredictable instabilities in the animals' instantaneous breathing rate require the operator to adjust the window and/or depth of anaesthesia throughout the scan to compensate. The use of automated data reacquisition, as described, adaptively tracks any changes in the respiration rate dynamically without operator intervention, and reduces motion corruption of data acquired at the entry to each breath to maximise image stability even in the presence of unstable physiology.

The R-R interval of an isoflurane anaesthetized normal healthy mouse generally lies well within 100–150 ms. The CR-gated acquisition block of duration 89.6 ms is therefore considered to be sufficiently short to be essentially unaffected by any normal changes in the heart rate.

The scan time for the stability data presented in Fig. 4, Fig. 5, Fig. 6, Fig. 7 increased from 11.5 s per frame for ungated imaging to about 21.1 s per frame with R-gating and reacquisition and about 25.2 s per frame with CR-gating and reacquisition, once the steady state had been established. The relatively small increase in scan time between R-gating and CR-gating is a result of the large proportion of the cardiac cycle that is used for imaging. There is a trade-off to be made between the proportion of the cardiac cycle that is used for imaging, and the number of heartbeats required to complete the scan, as the latter dictates the total scan time. We have previously observed that measurement of *T*_1_ in a number of organs have improved precision when acquiring data over a larger proportion of a relatively low number of heartbeats than when acquiring data over a smaller proportion of a relatively high number of heartbeats, as tested when acquiring 32 or 8 k-space lines per heartbeat at the same TR [19]. This suggests that long term physiological instabilities may adversely impact imaging performance. Some physiological motions such as peristalsis and bladder filling cannot be controlled with this gating technique and these will limit the image stability that can be achieved in regions experiencing these effects. In such cases imaging speed and minimum scan time dictate the achievable resolution.

The stability data for all the acquisition modes in four consecutively scanned mice are publically available courtesy of the Bodleian Digital Library Systems and Services of the University of Oxford at https://ora.ox.ac.uk/objects/uuid:28040887-8aac-49f6-9c8f-91ddcb0627a4. The data are in NIfTI-1.1 format (https://nifti.nimh.nih.gov/) and can be viewed with ImageJ (https://imagej.nih.gov/ij/). It is particularly instructive to inspect the stability of data by selecting a plane in the 3D volume and scrolling through the time course. It can readily be seen that the data presented here are entirely representative.

**Fig. 8:**
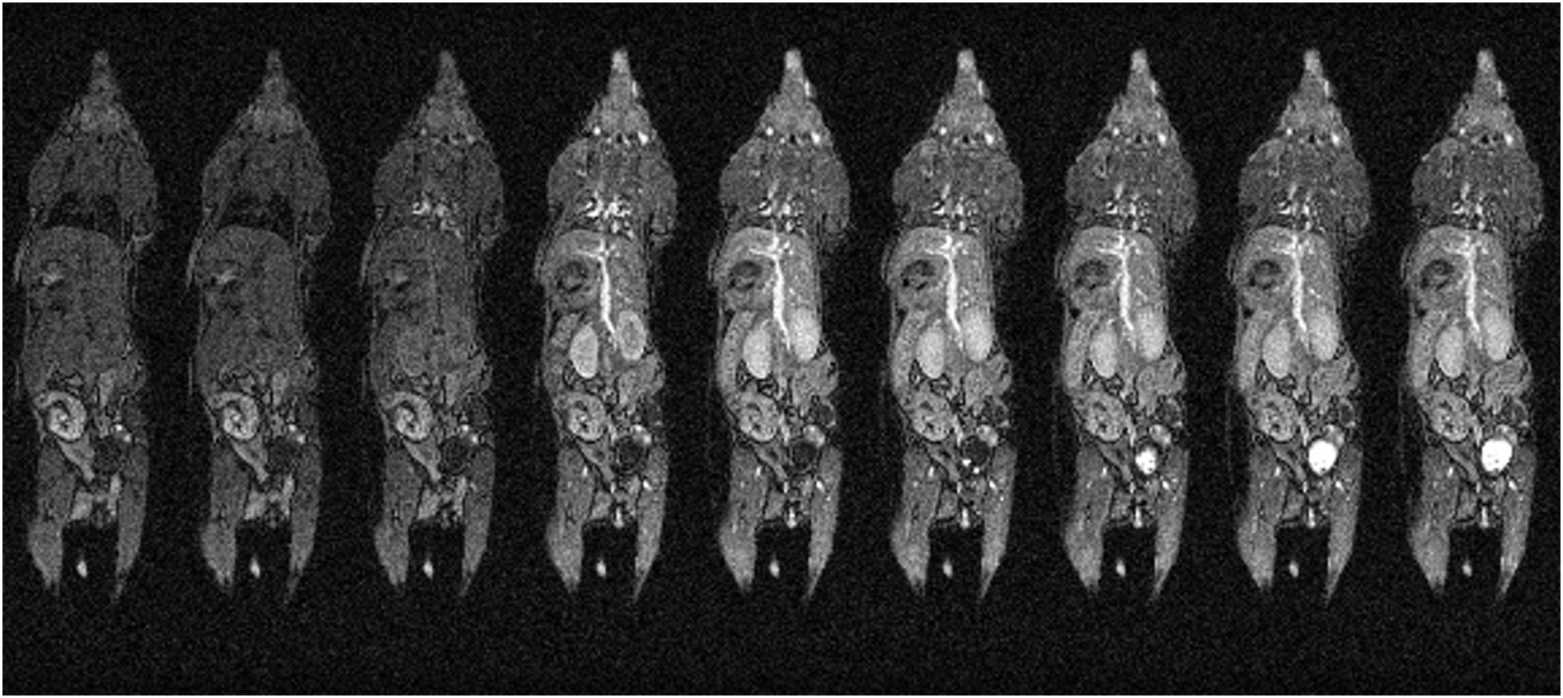
Coronal image slice from volumes 9, 10, 11, 12, 15, 20, 30, 40 and 50 taken from a 3D DCE-MRI time course. Gd-contrast agent was infused via a tail vein cannula over 15 s starting at the beginning of volume 11. 3D data volumes were acquired every 12–15 s.

A coronal slice taken from different frames of a whole body DCE-MRI scan are presented in Fig. 8. For the examination of tracer uptake kinetics, scan efficiency is absolutely critical. By acquiring 64 phase-encode steps within each heartbeat it was possible to acquire a complete image volume in 12–15 s, and to examine the early, though not purely the first pass, distribution of Gd tracer compounds over the whole body including in the lung and heart. A retrospective CR-gated scan would only be able to acquire a single phase-encode step with each heartbeat. To minimise the effects respiration motion to the same extent as presented here would therefore result in a 3D temporal resolution in the region of 12–16 min, which is far too long for DCE-MRI. The 3D temporal resolution of the prospectively CR-gated method presented here varies between 12 and 15 s according to the animals' instantaneous breathing rate.

The first image acquired post-delivery (volume 11) highlights the heart, lungs, and major vessels before significant tracer delivery is seen within the tissue bed (volume 12), and the uptake shows good anatomical fidelity as they are minimally corrupted through movement. Such high-fidelity images can only be acquired at this frame rate through the use of prospective gating in conjunction with automatic reacquisition of motion corrupted data as multiple k-space lines can be acquired within each heartbeat. Further accelerations may be possible using techniques such as partially parallel acquisitions and compressed sensing, and the use of these is independent of and compatible with, the gating control schemes described.

## 4 Conclusions

Prospective gating in conjunction with cardio-respiratory synchronisation, k-space segmentation and automatic reacquisition of respiratory motion corrupted data is demonstrated to enable highly efficient motion desensitised scanning in the mouse. Scans can be made more efficient than is possible with techniques such as retrospective gating and the examination of rapidly changing systems can be achieved.

## Figures and Tables

**Fig. 9:**
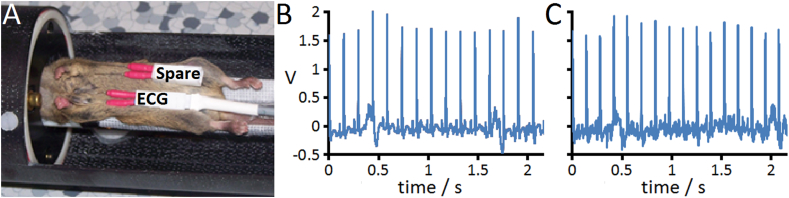
A: Positioning of ECG needles subcutaneously in the chest of a mouse. A spare detachable ECG probe head is added for illustration purposes. B: ECG trace from Mouse 1 recorded during the absence of scanning. C: ECG from Mouse 1 recorded during the rapidly switched strong magnetic field gradients of short TR 3D spoiled gradient echo imaging.

## Appendix A

ECG needle cables were cut 10 mm long, stripped, and the conductor attached to a crimp contact (JST BYM-001T-P0.6, Premier Farnell, UK). Pairs of contacts were inserted into RCY receptacles (JST SYR-02TV) to form ECG probe assemblies. A RCY plug (JST SYR-02TV-1) connects the needles to a Biopac ECG100C-MRI amplifier interfaced to a Biopac MP150 data acquisition system via a 2-core shielded cable (749-2541, RS Components Ltd., UK). Fig. 9 shows the typical positioning of needles and representative ECG traces in the absence and presence of rapidly switched strong magnetic field gradients.

The pressure balloon was attached to a pressure transducer (235-5762, RS Components Ltd., UK) and interfaced to the MP150 data acquisition system through a Biopac DA100C amplifier.

The respiration and ECG signals from the MP150 data acquisition system were fed to a Biopac DTU200 control unit to generate logic control signals according to adjustable threshold levels. The respiration logic signal output from the DTU200 has a duration determined by the threshold level. The signal was passed to a custom-built PIC-based device to generate a suitable R-logic gating signal with additional duration (τ in Fig. 1) set by a potentiometer to last until after each breath has completed. The 500 μs ECG C-logic signal output from the DTU200 is too short to use as a gating signal, so was passed to a custom-built 555 monostable timing unit (Maplin Electronics, UK) to generate a signal of suitable duration set by a potentiometer. The requirement for cardiac gating is that the signal duration is longer than the time between successive reads of the signal (typically the sequence TR), but shorter than the duration of an imaging block acquired in response to the gating signal (typically several TRs).

The ECG100C-MRI amplifier and DTU200 unit were set with minimum filtering to achieve good synchronisation with the cardiac R-wave. A 3 ms 5 V signal attenuated by 18 dB at the ECG needles was observed to generate a 1.5 V signal 7 ms in duration very similar to the observed in vivo ECG signals. The delay between the input 5 V signal and the output CR-logic signal varied between 3 and 6.5 ms depending on the DTU200 threshold setting.
